# Glucocorticoids coordinate the bladder peripheral clock and diurnal micturition pattern in mice

**DOI:** 10.1038/s42003-023-04464-3

**Published:** 2023-01-21

**Authors:** Ichiro Chihara, Hiromitsu Negoro, Jin Kono, Yoshiyuki Nagumo, Haruki Tsuchiya, Kosuke Kojo, Masanobu Shiga, Ken Tanaka, Shuya Kandori, Bryan J. Mathis, Hiroyuki Nishiyama

**Affiliations:** 1 Department of Urology, Faculty of Medicine, University of Tsukuba, Tsukuba, Ibaraki Japan; 2 Department of Urology, Graduate School of Medicine, Kyoto University, Sakyo, Kyoto, Japan; 3 Department of Urology, Tsukuba Medical Center Hospital, Tsukuba, Ibaraki Japan; 4 International Medical Center, University of Tsukuba Affiliated Hospital, Tsukuba, Ibaraki Japan

**Keywords:** Bladder, Biochemical networks, Bladder disease

## Abstract

Peripheral clocks function to regulate each organ and are synchronized though various molecular and behavioral signals. However, signals that entrain the bladder clock remain elusive. Here, we show that glucocorticoids are a key cue for the bladder clock in vitro and in vivo. A p*Bmal1*-dLuc human urothelial cell-line showed significant shifts in gene expression after cortisol treatment. In vivo, rhythmic bladder clock gene expression was unchanged by bilateral adrenalectomy but shifted 4 h forward by corticosterone administration at the inactive phase. Moreover, the bladder clock shifted 8–12 h in mice that underwent both bilateral adrenalectomy and corticosterone administration at the inactive phase. These mice showed decreases in the diurnal rhythm of volume voided per micturition, while maintaining diurnal activity rhythms. These results indicate that the diurnal rhythm of glucocorticoid signaling is a zeitgeber that overcomes other bladder clock entrainment factors and coordinates the diurnal rhythm of volume voided per micturition.

## Abstract (web summary)

The diurnal rhythm of glucocorticoid is shown to be a master regulator of the bladder clock in mice.

## Introduction

Circadian changes in functional bladder capacity, present in mammals^1–3^, result in decreases during the active phase and increases during the inactive phase^4–6^. This rhythm prevents frequent nocturnal awakenings, lengthening continuous sleep, along with regulation of circadian changes in urine production. Pathological conditions associated with disturbance of this rhythm (i.e., inadequate bladder capacity at night) are nocturnal enuresis and nocturia^7^, considered as immaturity or degeneration of the circadian bladder function, respectively^1,8^. Since both conditions decrease QOL^9,10^, and nocturia is associated with overall mortality^11,12^, clarification of bladder circadian rhythm on a molecular scale is required to fully explain the regulatory elements involved in urinary dysfunction.

Circadian rhythm in humans, based on an approximately 24-h cycle, is controlled by the central master clock located within the suprachiasmatic nucleus (SCN) that generates this rhythm through mechanistic transcription-translation feedback loops^13,14^. The core loop consists of the negative component of *PER* and *CRY* plus the positive component of *CLOCK* and *BMAL1* (also known as *ARNTL*)^15–17^. Oscillations of this core loop are followed by secondary, co-existing feedback loops involving *REV-ERB*, *ROR*, *DBP*, *EFBP4*, *TEF*, *HLF,* and others^18–20^. In addition to the whole-body circadian rhythm controlled by the central clock, each organ has its own functional circadian rhythm modulated by a peripheral clock^21–23^ which is itself regulated by several pathways, including autonomic nerves from the SCN, oscillating/circulating hormones, or indirect behavior regulation such as food intake, movement, and social contact^24–28^.

Of particular importance is the homeostatic hormone glucocorticoid (GC) that influences clock gene expression in several organs, including the kidneys^29,30^. Also bound to a 24-h circadian rhythm, synchronization of the SCN master clock with peripheral organ clocks relies on tightly regulated expression of GC coupled to GC receptors (GR) within the organs themselves^31^. More than 50 years have passed since the evening administration of GC was found to induce nocturia, studies of which focused on urine production rhythm but not bladder capacity^32^.

A peripheral clock exists in the urinary bladder, dependent on the oscillation of key timing genes^33–36^, but its molecular control mechanism is largely unknown. While a few reports assert that pathological conditions, such as hypertension or stress, may influence the disturbance of clock gene expression and that local receptor agonists (such as carbachol and ATP) alter the peak bladder clock hour in mice^37–39^, there are no reported studies on the specific signal(s) that can entrain the bladder clock. Herein, we show that glucocorticoids can both control the bladder clock and modulate diurnal voiding behavior in mice.

## Results

### Dexamethasone alters circadian clock rhythms in a human urothelial reporter cell-line

**Fig. 1:**
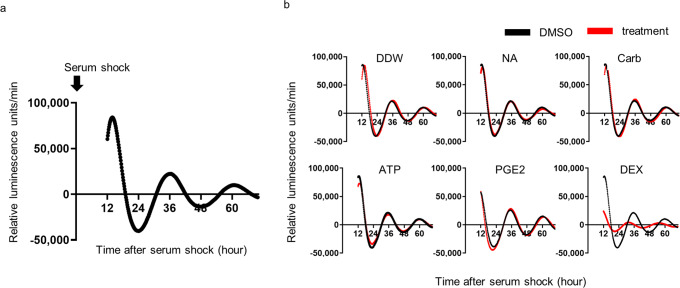
TRT-HU1 *Bmal1*-Luciferase cell luminescence and effect of reagents. **a** schematic diagram of the mean quantified *Bmal1*-Luciferase (*Bmal1*-Luc) relative luminescence intensities in 35 mm dishes after serum shock. **b** Successive changes of relative luminescence intensities of *Bmal1*-Luc cells by adding DDW, NA 10 μM, Carb 10 μM, ATP 10 μM, PGE2 10 μM, and DEX 0.1 μM to the medium, compared with 0.2% DMSO. One representative of two experiments with similar results is shown (DDW: distilled water, NA: noradrenaline, Carb: carbachol, ATP: adenosine triphosphate, PGE2: prostaglandin E2, DEX: dexamethasone).

To investigate the physiological signals that regulate the bladder clock, we first generated TRT-HU1 *Bmal1*-Luc cells to monitor continuous *Bmal1* expression in immortalized human urothelial cells. After synchronizing the cell clock by serum shock (SS) using 50% FBS, the influence of candidate substances on the rhythmic bioluminescence under control of TRT-HU1 was measured (Fig. 1a). Noradrenaline (NA), carbachol (Carb), ATP, prostaglandin E2 (PGE2), and dexamethasone (DEX) were chosen as candidates based on reports of their influence over clock genes. NA is a sympathetic neurotransmitter whose adrenergic signaling has been reported to affect clock gene expression in liver and vascular smooth muscle cells^40,41^ while Carb and ATP (agonists of muscarinic and purinoreceptors, respectively) have been reported to affect the expression of the *Per2* clock gene in the bladder^39^. PGE2 is a pro-inflammatory compound known to cause phase shifts in *Per1* expression rhythms in the liver, kidney, and heart^42^. Finally, DEX is an artificial compound with strong GC effects on clock gene expression in multiple organs and tissues but relatively unknown effect on the bladder^31^.

We found that the rhythm and amplitude of cell luminescence did not change after treatment with NA, Carb, ATP, or PGE2, but only with DEX. Furthermore, DEX caused the amplitude of cell luminescence to significantly decrease, shift the peak forward 10 h, and reduce the period to 22 h from 24 h (Fig. 1b). These results suggest that GC is a potential agent that can control the circadian expression of *Bmal1* in human urothelial cells.

### Glucocorticoids modulate clock gene expression in a human urothelial cell-line

**Fig. 2:**
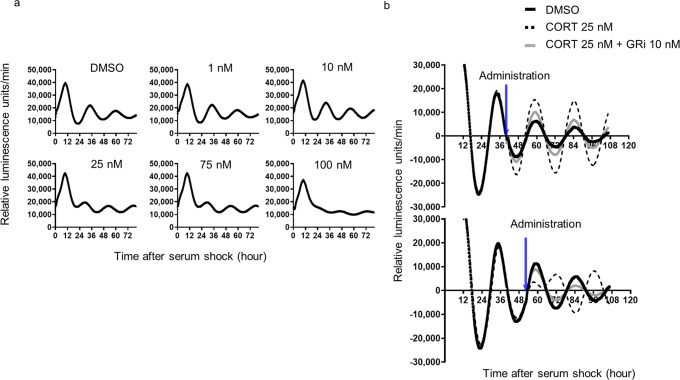
*Bmal1*-Luc cell luminescence intensities with cortisol addition. **a** Successive changes of luminescence intensities of *Bmal1*-Luc cells by adding 1 nM, 10 nM, 25 nM, 75 nM, and 100 nM cortisol to the medium. One representative of two experiments with similar results is shown. **b** Successive changes of relative luminescence intensities of *Bmal1*-Luc cells by administrating 25 nM cortisol and glucocorticoid receptor inhibitor (GRi) at 40 h and 52 h after serum shock (Blue arrows). One representative of two experiments with similar results is shown.

To investigate molecules that regulate the circadian rhythm of *Bmal1* expression in the bladder under physiological conditions, further studies were conducted using cortisol, which is a physiologically relevant, constitutively expressed GC. First, various concentrations of cortisol were applied to TRT-HU1 *Bmal1*-Luc cells to determine the dose-dependent effect of GC on the *Bmal1* expression rhythm. We observed that the amplitude of luminescence decreased after 25 nM and was further reduced at 75 nM and 100 nM (Fig. 2a). Given that the peak free cortisol concentration, as encountered in daily clinical practice, is usually 20–30 nM in humans^43^, this result indicates that GC affects *Bmal1* expression in a dose-dependent manner. In humans, the serum cortisol level has a circadian rhythm, with low levels at night, that rises in the early morning, reaches a peak upon waking, and declines over the course of the day to low levels in the evening^44,45^. *Bmal1*, conversely, peaks at night and troughs at noon^46^.

To investigate the relationship between this circadian rhythm of serum cortisol level and that of urothelial clock genes, we administered 25 nM of cortisol to the TRT-HU1 *Bmal1*-Luc cells at both a physiological timing (PT) and an opposite timing of the physiological peak (non-physiological timing: NPT). Here, based on the expression rhythm of TRT-*Bmal1*-Luc cells, we defined PT as 40 h after and NPT as 52 h after serum shock. Cortisol administration at PT increased the amplitude but did not influence the phase of the luminescence rhythm; however, cortisol administration at NPT reversed the phase of the luminescence rhythm (Fig. 2b). These results suggest that the cortisol peak timing is important for controlling *Bmal1* expression rhythms in urothelial cells. These changes in phase and amplitude induced by cortisol were inhibited by glucocorticoid receptor inhibitor (GRi) mifepristone at 10 nM, administered simultaneously with cortisol (Fig. 2b). This suggests that the effect of cortisol on *Bmal1* expression in urothelial cells is GR mediated in a mineral corticoid-independent fashion. Additionally, GCs can regulate the circadian rhythmic expression of *Bmal1* via the GR signaling pathway in a concentration- and time-dependent manner in a human urothelial cell-line.

### GC regulates the bladder clock in vivo

**Fig. 3:**
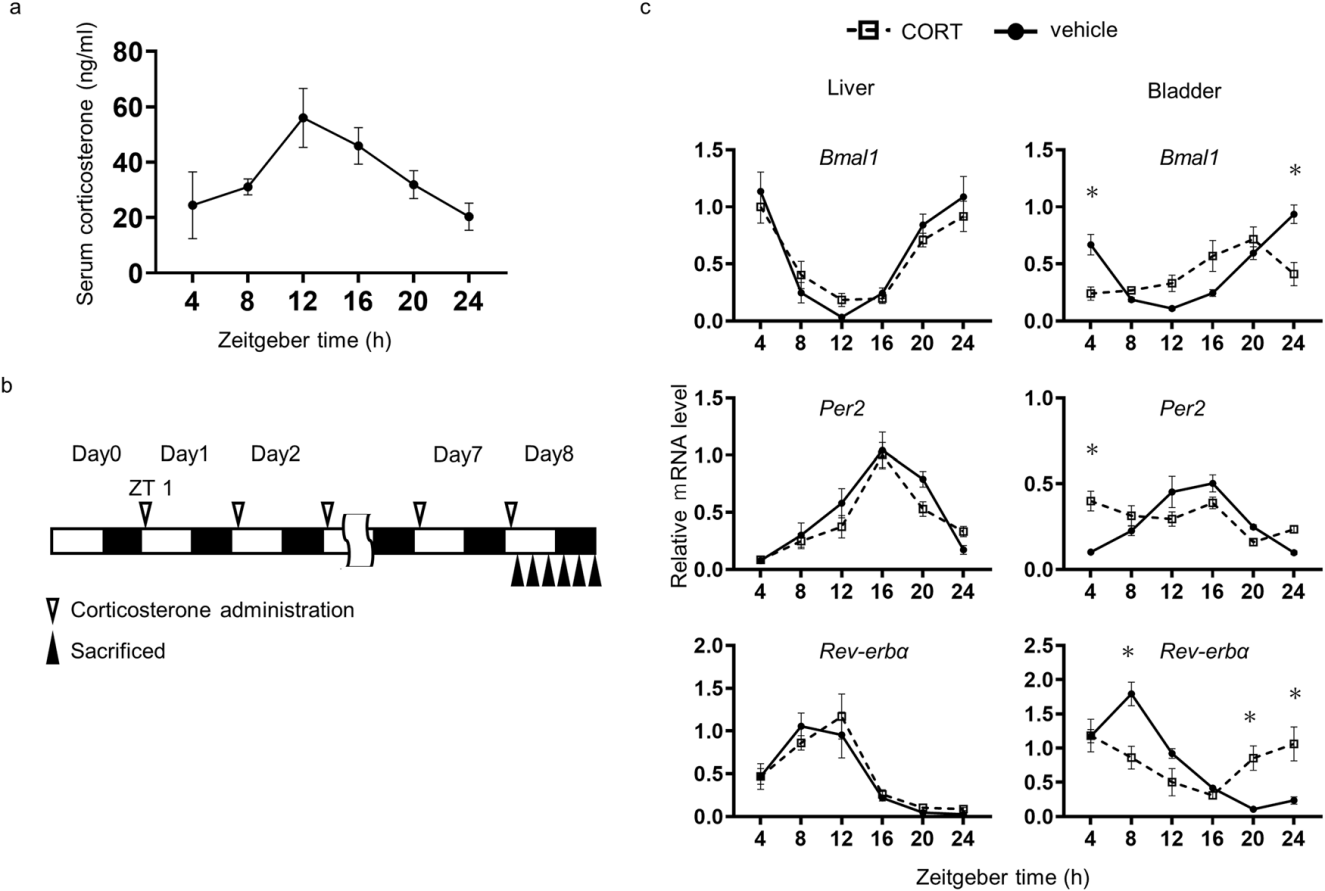
Effect of corticosterone administration at the non-physiological timing. **a** Diurnal change in serum corticosterone levels in normal mice at our facility. Each data series represents the mean ± SEM and 4 mice per time point. **b** A schematic diagram of the experiment that mice were orally administered corticosterone (CORT) for 7 consecutive days at ZT 1 and sacrificed every 4 h on day 8 (ZT 4,8,12,16,20,24). **c** Relative mRNA accumulation of *Bmal1*, *Per2,* and *Rev-erbα* in the liver and bladder from mice vehicle (solid line) and CORT (dash line) at ZT 1. Each data series represents the mean ± SEM and 5 mice per time point. Differences were determined by two-way ANOVA and Sidak’s multiple comparisons test. *p < 0.01 v.s. vehicle (ZT: Zeitgeber time).

We next conducted in vivo experiments using mice and corticosterone (CORT), the chief murine GC. Serum CORT levels peaked at ZT12, just before the active phase onset (Fig. 3a), comparable to previous reports^47,48^. To investigate whether CORT affects expression of clock genes in the bladder, it was administered to mice at NPT of ZT1, just after the onset of sleep, for 7 consecutive days and the mice were sacrificed every 4 h (Fig. 3b). In this case, serum CORT levels were elevated at ZT4 to a similar level as the physiological peak, while the peak level of the CORT group did not differ from vehicle (Supplementary Fig. S1a). In the liver, there were no phase shifts or amplitude changes in *Bmal1*, *Per2*, or *Rev-erbα* expression, except for a slightly lower peak in *Baml1* expression in the CORT group compared to the vehicle group. On the other hand, in the bladder, the peak expression of *Bmal1* and *Rev-erbα* shifted forward 4 h (Fig. 3c). This indicates that the bladder clock is more susceptible to CORT than the liver.

**Fig. 4:**
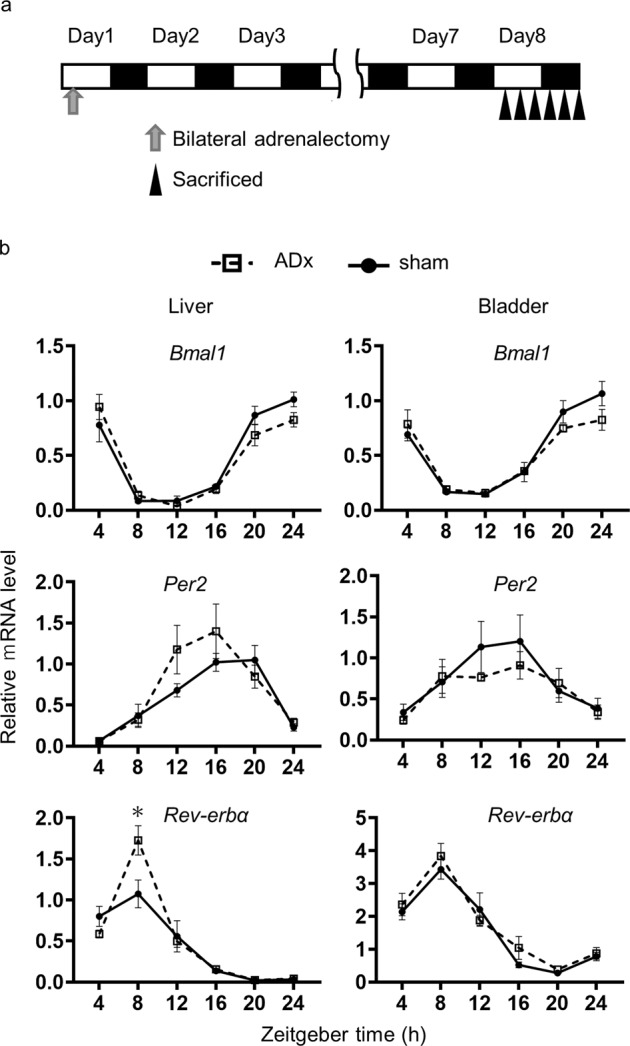
Effect of bilateral adrenalectomy. **a** A schematic diagram of the experiment that bilateral adrenalectomy (ADx) performed on Day 1 and sacrifice death on Day 8. **b** Relative mRNA accumulation of *Bmal1*, *Per2,* and *Rev-erbα* in the liver and bladder from sham (solid line) and ADx (dash line). Each data series represents the mean ± SEM and 5 mice per time point. Differences were determined by two-way ANOVA and Sidak’s multiple comparisons test. *p < 0.01 v.s. sham.

To further examine the effect of CORT rhythm on the bladder clock, we applied a mouse model in which the bilateral adrenal glands were removed (ADx) and sacrificed them after a week (Fig. 4a). The serum level of CORT was significantly decreased, but not completely eliminated, at the physiological peak timing of both ZT12 and ZT16 in ADx mice compared to sham mice (Supplementary Fig. S1b). Corticosterone is known to be secreted by the brain, salivary glands, and skeletal muscles in addition to the adrenal glands. Hence, even if bilateral adrenal glands are removed, the remaining amounts secreted prevent death^49–51^. There was little influence of ADx on the expression levels and rhythms of *Bmal1*, *Per2*, or *Rev-erbα* in both the liver and the bladder except that the peak of *Rev-erbα* expression increased significantly in the liver of ADx mice compared to sham mice (Fig. 4b).

**Fig. 5:**
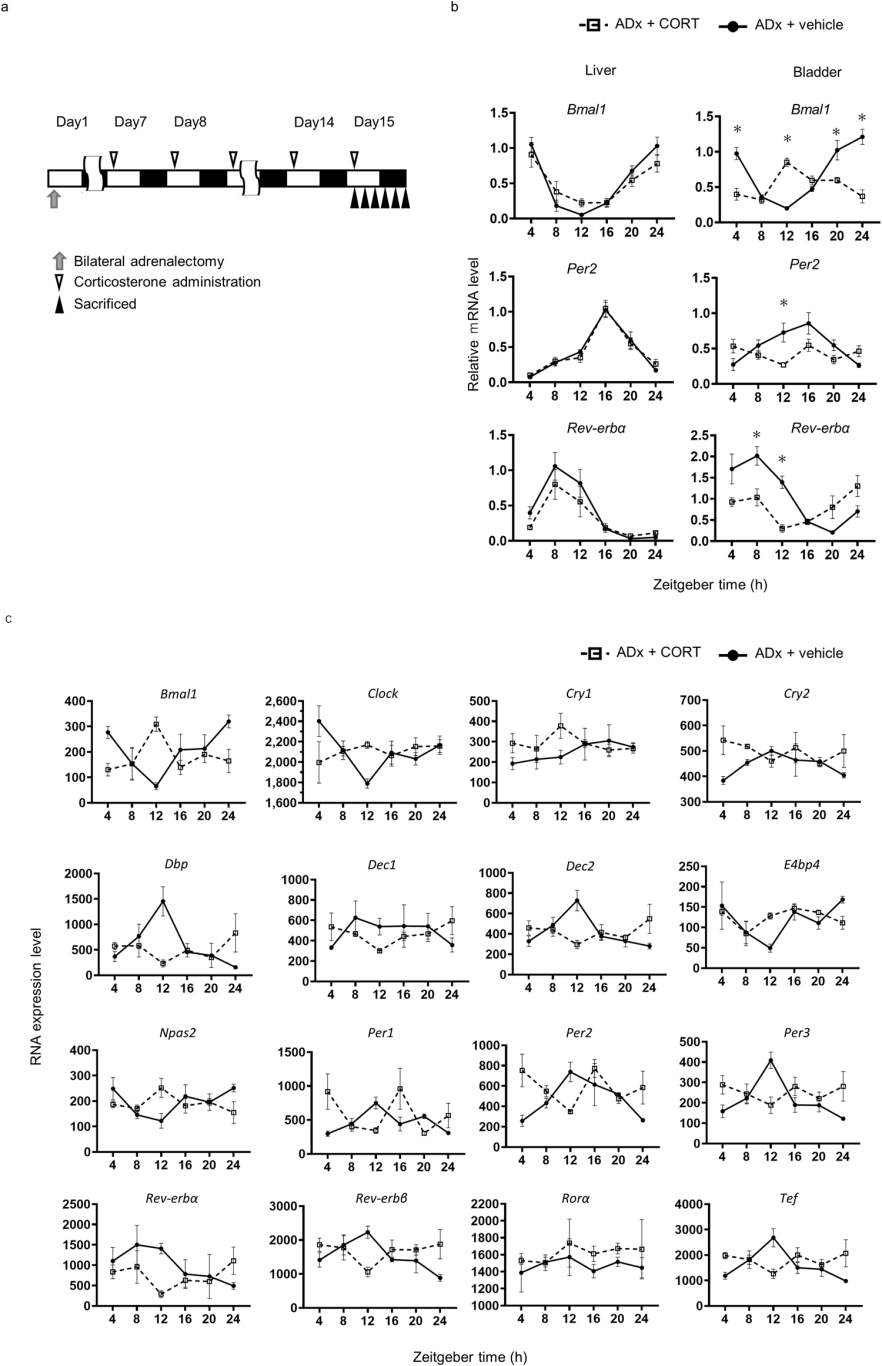
Effect of bilateral adrenalectomy and corticosterone administration at the non-physiological timing. **a** A schematic diagram of the bilateral adrenalectomy (ADx) performed on Day 1 and corticosterone administered orally for 7 days at ZT 1 from Day 8 after ADx and sacrifice on Day 15 (ADx + CORT). **b** Relative mRNA accumulation of *Bmal1*, *Per2,* and *Rev-erbα* in the liver and bladder from ADx + vehicle (solid line) and ADx + CORT (dash line). Each data series represents the mean ± SEM and 5 mice per time point. Differences were determined by two-way ANOVA and Sidak’s multiple comparisons test. *p < 0.01 v.s. ADx + vehicle. **c** Temporal mRNA accumulation of clock genes in the bladder from ADx + vehicle (solid line) and ADx + CORT (dash line) by analyzing RNA-seq. Each data series represents the 3 mice per time point.

Next, we administered CORT at ZT1 of NPT for 7 consecutive days after a week of ADx and created a mouse model with an opposite serum CORT level peak (ADx + CORT) to compare to a vehicle control model (ADx + vehicle). (Fig. 5a). In the liver, little change was observed in the expression rhythms of *Bmal1*, *Per2*, or *Rev-erbα*, except that a significant decrease of amplitude was detected in *Rev-erbα* (Fig. 5b). On the other hand, the expression patterns of these genes were markedly disturbed in the bladder as the peak and trough timing of *Bmal1* switched inversely, the expression rhythm of *Per2* was unclear, and the peak of *Rev-erbα* expression decreased and shifted 8 h forward (Fig. 5b). Triamcinolone, a synthetic steroid with only glucocorticoid action and little mineralocorticoid action^52^, was administered to ADx mice in place of corticosterone at the same titer. In the bladders of ZT12 mice, as in ADx + CORT, *Bmal1* expression increased and *Per2* and *Rev-erbα* expressions decreased. Also, in the liver, similar to ADx + CORT, only *Bmal1* increased, but *Per2* and *Rev-erbα* remained unchanged (Supplementary Fig. S2). To confirm the influence of opposite diurnal CORT rhythms on the network of clock genes in the bladder, RNA-seq of the bladder in these two groups was performed. Most clock genes of interest tended to either advance the peak shifting 8 to 12 h forward or disrupted diurnal rhythms (Fig. 5c and Supplementary Table S1a). These data demonstrate that GC can regulate the expression rhythm of clock genes in the bladder tissue in vivo.

### GC affects diurnal micturition patterns in mice

**Fig. 6:**
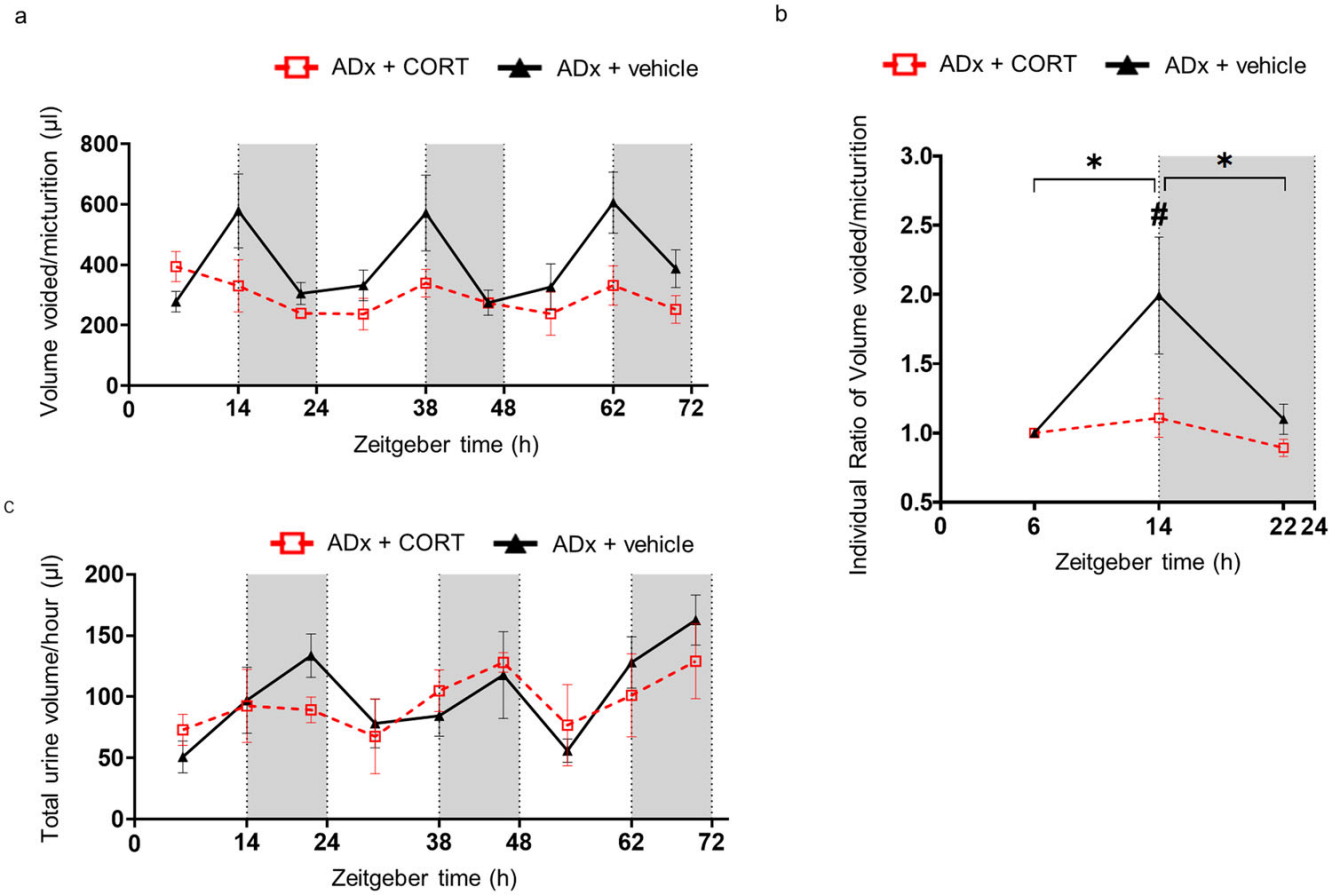
The diurnal rhythm of micturition. **a** Volume voided per micturition per 8-h averages measured on 3 consecutive days in ADx + vehicle (solid black line) and ADx + CORT (dash red line). Each data series represents the mean ± SEM and 5 mice per time point. **b** Average of 3 days results of measured volume voided per micturition average per 8-h in ADx + vehicle (solid black line) and ADx + CORT (dash red line). Each data series represents the ratio of ZT14, 22 to ZT6 as 1 in the same and the mean ± SEM and 5 mice per time point. Differences were determined by two-way ANOVA and Sidak’s multiple comparisons test. ^#^*p* < 0.01 v.s. ADx + vehicle and Dunnett’s multiple comparisons test. *p < 0.01 v.s. ZT 14 in ADx + vehicle and ADx + CORT. **c** Total voided volume per h per 8-h averages measured on 3 consecutive days in ADx + vehicle (solid line) and ADx + CORT (dash red line). Each data series represents the mean ± SEM and 5 mice per time point.

To investigate whether the non-physiological peak of GC rhythms can affect the diurnal rhythm of volume voided per micturition in mice, a free-micturition pattern was measured for 3 consecutive days in ADx + CORT and ADx + vehicle mice using the aVSOP method. The ADx + vehicle mice had a normal diurnal rhythm with the volume voided per micturition decreasing during the active period and increasing during the inactive period (Supplementary Table S2). In contrast, ADx + CORT mice did not display a similar diurnal rhythm (Fig. 6a). A significant increase in ADx + vehicle was observed at the ZT14 average of measured volume voided per micturition over 3 consecutive days, which was determined by two-way ANOVA and Tukey’s multiple comparisons test (Fig. 6b). Other control groups, such as vehicle, CORT, and sham, maintained their diurnal rhythms of volume voided per micturition (Supplementary Fig. S3, Supplementary Table S3). Interestingly, the rhythm of total urine volume per hour was maintained in both groups, increasing during the active phase and decreasing during the inactive phase in both groups (Fig. 6c). Furthermore, the rhythm of activity, measured by exercise wheel spins, was also maintained in both groups, with increases during the active phase and decreases during the inactive phase, while the dark phase rotations increased in ADx + CORT mice (Supplementary Fig. S4). Although alterations in urine production or activity rhythms could have influenced the volume voided per micturition^53,54^, ADx + CORT mice maintained a diurnal rhythm of urine production and activity, decreasing only in volume voided per micturition. These results, therefore, demonstrate that the loss of the diurnal rhythm of volume voided per micturition in ADx + CORT mice was an independent phenomenon distinct from the rhythms of urine production or activity. Collectively, the present study demonstrates that GC rhythm coordinates the diurnal rhythm of bladder capacity.

### Global transcriptomic analysis of murine diurnal rhythm genes using RNA-seq

**Fig. 7:**
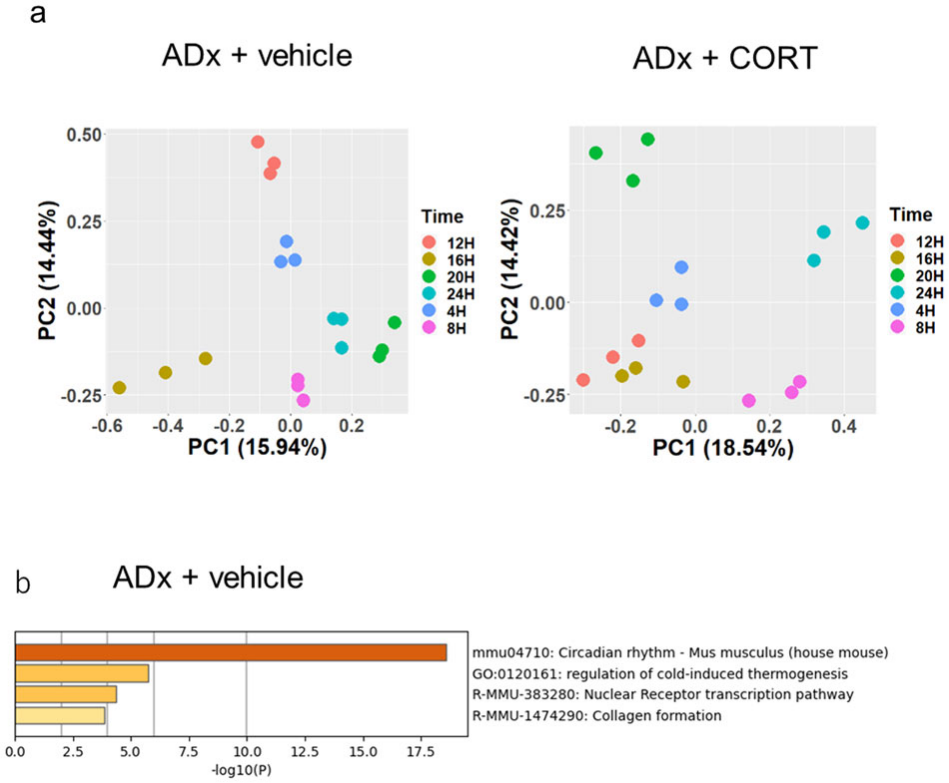
Global transcriptome analysis using RNA-seq in ADx + vehicle and ADx + CORT mice. **a** PCA plots with *N* = 3 at ZT 4, 8, 12, 16, 20, and 24 in the ADx + vehicle and ADx + CORT groups, respectively. **b** Pathway analysis was performed using Metascape for the diurnal rhythmicity genes (defined as greater than the MaxCorr of 0.85 from the cosine curve with a 1.5-fold amplitude of expression level) in the ADx + vehicle group.

**Fig. 8:**
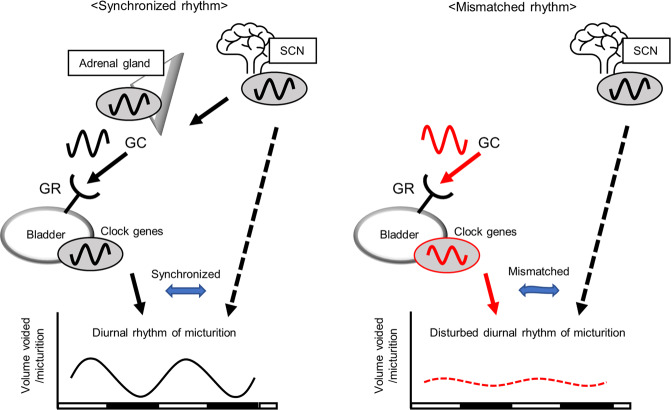
A schematic diagram of this study. The present study suggests that glucocorticoid secreted by the adrenal cortex acts on the glucocorticoid receptor in the bladder to be an entrainer of the bladder clock and that a mismatch between the central and the peripheral bladder clocks decrease the diurnal rhythm of volume voided per micturition. (GC: glucocorticoid, GR: glucocorticoid receptor).

To further investigate the diurnal rhythm transcriptome, we performed an enriched analysis of RNA-seq data from our ADx + CORT and ADx + vehicle mouse models. Differentially expressed genes (fold change > 2.0, *p* < 0.05) in the ADx + CORT (226 genes) and ADx + vehicle (215 genes) groups were extracted and analyzed by PCA plots, which showed that RNA expression profiles were similar for each duplicated time point but had time-specific distributions within each group (Fig. 7a). Diurnally expressing genes with high fit rates to the cosine curve and 1.5-fold in amplitude were extracted^30^, revealing 29 genes in the ADx + vehicle group (Supplementary Table. S1b) but no gene in the ADx + CORT group. These results indicate that non-physiological peak GC can significantly shift the rhythmic expression of clock genes in the bladder as shown in Fig. 5b, even if entrainment is not entirely accomplished. Annotation of these 29 genes of interest in the ADx + vehicle group confirmed that circadian rhythm pathway was found to be highly changed (Fig. 7b). Taken together with the ADx + vehicle mice that maintained rhythmic expression of circadian clock genes in the bladder, we concluded that CORT signaling is not the sole regulator of clock rhythm and that central signaling or other factors are likely involved in maintenance of the bladder clock. From these observations, we propose that GCs are an entrainer of the bladder clock and a mismatch between the central and the peripheral bladder clocks decrease the diurnal rhythm of volume voided per micturition (Fig. 8).

## Discussion

Peripheral clocks cross-interact with other peripheral clocks by means of inter-organ networks to maintain circadian homeostasis but the tuning mechanisms in each organ differ. Such mechanisms have been elucidated in some organs, such as the liver, lungs, and heart, but control paradigms within the bladder remain elusive^26^.

In the present study, dexamethasone and the glucocorticoid cortisol significantly decreased *Bmal1*-Luc signal in a transfected, human urothelial cell-line but NA, Carb, ATP, or PGE2 had little effect. The influence of GC on the cells was both concentration and time-dependent, readily inhibited by mifepristone, and consistent within groups. Cortisol administration at PT after serum shock, at comparable elevated blood cortisol levels in humans, increased the amplitude while cortisol administration at NPT reversed the phase of the rhythm. In vivo, the ADx mice had little change in bladder clock gene expression compared with the sham mice but the CORT mice (administration of corticosterone at non-physiological timing) and the ADx + CORT mice had significant alterations in the diurnal rhythm of clock gene expression compared to controls. We observed that *Bmal1* and *Rev-erbα* peaks shifted forward 4 h in the CORT mice and, of note, 8–12 h forward in the ADx + CORT mice. Moreover, RNA-seq revealed that most clock gene peaks shifted forward 8 to 12 h in the ADx + CORT mice, indicative of changes in the diurnal rhythm of volume voided per micturition, but all mice groups maintained a normal rhythm. These results indicate that the diurnal GC rhythm is involved in both entrainment of the bladder clock and coordination of the diurnal rhythm of volume voided per micturition.

The key finding of this study is that glucocorticoids act as an entraining signal to the bladder clock genes, generally in line with the few published reports showing that certain substances or pathological conditions influence clock gene expression in the bladder. However, discrepancies between our results and published reports remain. Wu et al. reported that the peak of *Per2* in the bladder shifted by applying Carb and ATP^39^ while the present study did not. We hypothesize that these observed differences stem from experimental design variances in addition to the differences in the targeting strategies for the clock genes *Bma11* and *Per2*. Since Wu et al. directly studied bladders of *Per2*-Luc knock-in mice in vivo and the present report studied Carb and ATP in urothelial cells in vitro, we assert that Carb and ATP may have exhibited off-target effects in smooth muscle cells, myofibroblasts, or indirectly to the bladder clock. In contrast, Ihara et al. reported that mice experiencing 2-h restraint stress (RS) during the light (inactive) phase (RS mice) had disrupted rhythms of *Bmal1* and *Per2* and a shift forward in the peak time of *Rev-erbα*, resulting in abolished diurnal differences in urine volume voided per micturition^38^. Since, in that report, the RS would also be expected to precipitate stress-induced GC secretion^55^, that result is comparable to our findings that the CORT alone group experienced forward peak shifting of *Bmal1* and *Rev-erbα* (by 4 h) and attenuation of *Per2* rhythmicity, indicating that GC can modify the rhythm of bladder clock genes. On the other hand, the day-night rhythm of micturition was lost after day 3 in the RS mice but was maintained in our CORT alone group even after day 7. One possible explanation for these differences is that the psychological impact of RS could influence urinary behavior in a way not directly associated with GCs, since stress affects the lower urinary tract at various stages from the brain to the bladder^56^. The other possibility is that the induced stress affected the central clock, which might result in a disrupted day-night micturition rhythm. Chronic stress is known to alter SCN rhythm homeostasis^57^ separately from GC due to minimal GR expression in the SCN^29^.

The other key finding in the present study is that the day-night rhythm of micturition remained in the groups of CORT alone and ADx + vehicle but disappeared in the ADx + CORT group. This difference can be explained from the viewpoint of the effects of central and bladder clocks on the diurnal rhythm of micturition. In the CORT alone group, the effect of GC administration at non-physiological timing on the bladder clock was an only 4-h shift in the phase peak while, in the ADx + vehicle, eliminating the GC signals had little influence on the bladder clock. As these bladder rhythms would not largely differ from their central rhythms, the diurnal voiding pattern could therefore continue the original rhythm. On the other hand, in the ADx + CORT mice, the bladder clock phase shifted almost 12 h, which significantly conflicted with the central rhythm. Large asynchronization effects could result in the loss of diurnal micturition rhythms. This desynchronization can be caused clinically by evening steroid administration in patients with adrenocortical insufficiency, but chronic stress conditions may also be causative. To maintain the diurnal micturition rhythm, it is important to prevent mismatch between the central and peripheral bladder clocks rhythms. However, the power balance between the bladder and central clock remains unclear since the max correlation rate (MaxCorr) of the most of the canonical clock genes in the bladder decreased in the ADx + CORT group (Supplementary Table 1a), indicating that the bladder clock was not completely functional after the 12-h phase shift. In addition, since there was no gene that showed significant diurnal variation from RNA-seq analysis in the ADx + CORT group, we concluded that diurnal variation in bladder function is not established at the transcriptional level. With this possibility in mind, if the bladder clock shifts by 12 h but maintains downstream control, the diurnal rhythm of volume voided per micturition might also shift 12 h even if it conflicts with the central clock. Further research is needed to elucidate the relationship between central and peripheral controls with respect to bladder function.

Peripheral clocks are known to have organ specificity with respect to their primary entraining signals. For example, in the liver, Reznick et al. reported that the hepatic clock phase shifts 6–12 h by feeding at inactive phase^58^, suggesting that feeding is a strong time cue for the liver clock. In the present study, we compared the liver to the bladder and performed GC stimulation at ZT1. This was based on a report by Tahara et al. which showed the differential effects of restraint stress at ZT0-2 on peripheral organs in mice^59^, namely an anti-phasic rhythm in the submandibular gland, dampened or nullified rhythm in the kidney, and unaltered in the liver. Since GC was known to increase markedly during stress loading^60,61^, we speculated that GC stimulation at ZT0-2 involved the modulation of GC-sensitive peripheral clocks but not the liver clock. Sujino et al. also compared the organ specificity of clock regulation by GC in kidneys, lungs, and the liver, reporting that GC functioned as a strong zeitgeber in the kidneys and lungs, but was inferior to feeding in the liver^30^. Consequently, these findings are consistent with the results of the present study in that GC stimulation at ZT1 had influence on the bladder clock but little on the liver one. Our study had some limitations. First, the periodicity of clock genes was measured during a single cycle and light-dark conditions only, not under constant dark conditions. Two or more cycles would have been desirable to enhance the resolution of periodicity changes in the bladder clock but, instead, our RNA-seq analysis of multiple genes found that such changes were not discrete alterations in individual clock genes but rather multiple genes interacting within the bladder. Regarding the effects of light exposure, since we focused on the peripheral clock in this study, we utilized only light-dark cycles consistent with clinical conditions. Second, our mRNA-level examination did not fully account for possible mechanisms of posttranscriptional regulation, such as protein expression or phosphorylation. Because RNA-seq showed that bladder clock genes were rhythmically expressed in ADx + vehicle mice but genes known to be involved in urinary function and fluctuate diurnally (such as *Cx43*, *Piezo1,* and *TRPV4)* were not (Supplementary Fig. 5), GC, through its influence on gene expression or transcriptional regulation, may be necessary for downstream genes to secure a proper diurnal rhythm. Third, the possibility of GC influence on sleep disturbance cannot be ruled out since electro-encephalography was not conducted. However, even if the exercise wheel count was not fully representative of sleep-wake cycles in a strict sense, the CORT and ADx + vehicle mice maintained diurnal activity rhythms and diurnal changes in volume voided per micturition, suggesting that the GC administration itself was not grossly disruptive to sleep-wake patterns. Finally, we did not assess water intake, which may correlate with the amount of urine production and thus the volume voided per micturition^54^. In our case, we calculated the amount of urine per hour by dividing the amount of micturition by the elapsed time and substituted this for the amount of urine production (Fig. 6c). Since the urine production rate did not differ between ADx + CORT and ADx + vehicle groups, we believe that urine production rates had little influence on the observed differences in volume voided per micturition.

The identification of the previously unknown bladder clock entrainer may provide clues to detect further mechanisms by which GC regulates the bladder clock and to elucidate the physiology and pathophysiology of the bladder clock. In addition, since GC was found to be a factor that influences the diurnal rhythm of volume voided per micturition, abnormalities in GC signaling and the bladder peripheral clock may be involved in the pathogenesis of nocturia and nocturnal enuresis. Future research is expected to establish new treatment methods with regard to these factors.

In conclusion, the present study demonstrates that the diurnal rhythm of GC signaling is involved in the control of the bladder peripheral clock and a mismatch between the central and peripheral bladder clocks decrease the diurnal rhythm of volume voided per micturition. Clarifying more details of the regulatory mechanisms within the peripheral bladder clock would help us understand the pathogenesis of nocturnal enuresis and nocturia that could lead to new therapeutic approaches.

## Methods

### Cells and cell culture

The hTERT-immortalized human urothelial (TRT-HU1) cell-line was used in this study, as previously reported^62,63^. The p*Bmal1*-dLuc lentiviral reporter was a kind gift from Prof. Andrew C. Liu (Department of Biological Sciences, University of Memphis, Tennessee, USA). TRT-HU1 reporter cell-lines stably expressing p*Bmal1*-dLuc (*Bmal1*-Luc) were generated using a previously reported transduction protocol for lentivirus-mediated gene delivery^64^. TRT-HU1 and *Bmal1*-Luc cells were maintained in Dulbecco’s modification of Eagle’s medium (DMEM; Fujifilm Wako, Japan) with 15% FBS (Biosera, USA), non-essential amino acids (Thermo Fisher Scientific, USA), and 1.15 mM 1-thioglycerol in a humidified atmosphere of 5% CO_2_ at 37 °C, as previously described^62^. Cultures were not routinely tested for mycoplasma contamination.

### Bioluminescence recording

To monitor cellular, autonomous circadian rhythms, *Bmal1*-Luc cells were plated in 35 mm plastic dishes (1.4 × 10^5^ cells per dish, 8 dishes per experiment). Luciferin (VivoGlo; Promega, Madison, WI) was added to the culture medium to a final concentration of 0.1 mM. The recording chamber of a Kronos-Dio luminometer system (Model AB-2550, ATTO Co.) was set at 37 °C for the duration of the experiment. The photon counting time was 1 minute per measurement with a sampling interval of 10 min. Bioluminescence was continuously monitored without interruption for 3–5 days.

### Serum shock analysis of hTERT-human urothelial cells

The TRT-HU1 cells were cultured until sub-confluent in DMEM with 15% FBS, followed by 48 h incubation in DMEM with 0.5% FBS. The cells were then treated with 50% FBS in DMEM for 2 h and then maintained in DMEM with 0.5% of FBS. Time 0 (hour) was set as the time point before serum shock.

### Chemicals

Mifepristone was obtained from Tokyo Chemical Industry, Co., Ltd. (Tokyo, Japan). Hydrocortisone (cortisol) was obtained from Fujifilm Wako (Osaka, Japan). Noradrenaline, ATP, and dexamethasone were obtained from Sigma Aldrich (Saint Louis, MO, USA). Carbachol was obtained from Nacalai Tesque Inc. (Kyoto, Japan). Prostaglandin E2 was obtained from Cayman Chemical Co. (Ann Arbor MI, USA). All chemicals used were of analytical grade.

### Real-time quantitative RT-PCR analysis

Total RNA extraction and complementary DNA synthesis from mouse bladders or cultured cells were accomplished using NucleoSpin® RNA (Takara Bio, Shiga, Japan) and High-Capacity cDNA Reverse Transcription kits (Thermo Fisher Scientific, USA). Real-time quantitative RT-PCR was performed with SYBR Green PCR Master Mix (Thermo Fisher Scientific, USA) and QuantStudio 5 (Thermo Fisher Scientific, USA). The thermal cycling conditions were 95 °C for 1 s and 60 °C for 20 s. Values were adjusted relative to the expression levels of the 18S ribosomal housekeeping gene and the ΔΔCt method was used to determine the relative gene expression of the genes of interest. The oligo sequences of the primers are listed in Supplementary Data.

### Animals

Eight-week-old male C57BL/6 mice were purchased from Charles River Laboratories Japan (Yokohama, Japan). All animals were placed in plastic cages under constant temperature (23.5 °C) with a 14-h light/10-h dark (L/D) cycle (lights-on at 5:00 am and Zeitgeber time [ZT] 0). All animal care and experimental procedures were performed in accordance with national and regional legislation on animal protection and all animal procedures were consistent with the University of Tsukuba’s Regulation of Animal Experiments. All experiments were approved by the Institutional Animal Care and Use Committee, University of Tsukuba (approval #20–375).

### Surgical procedures

Mice underwent a bilateral adrenalectomy (ADx) under anesthesia with isoflurane inhalation. Controls underwent a sham surgery in which bilateral skin incisions were made under anesthesia to expose the adrenal glands before closing. Both ADx and sham-operated mice were allowed to recover for 1 week postoperatively. All animals had free access to 1% NaCl solution during the recovery week.

### Serum collection and corticosterone measurement

Blood was immediately collected from mouse hearts after sacrifice and centrifuged to extract serum. Once serum was purified using methylene chloride dichloromethane, it was evaluated for corticosterone concentration using an ELISA kit (minimum sensitivity, 8.2 pg ml^−1^, Corticosterone ELISA Kit; Cayman Chemical Company, USA) and a microplate reader (Varioskan LUX; Thermo Fisher Scientific, USA).

### Drug administration

Corticosterone (Fujifilm Wako, Japan) at 0.2 mg/body was dissolved in 0.1 ml of 0.5 w v^−1^% methylcellulose 400 solution (Fujifilm Wako, Japan) and administered orally. In the control group, only 0.1 ml of vehicle was administered.

### Sampling

All mice were anesthetized with isoflurane and cardiac blood sampling was performed. The bladder and liver were then quickly dissected and immersed in RNA later (Invitrogen RNA later Stabilization Solution; Thermo Fisher Scientific, USA).

### Micturition analysis

As previously reported^34^, micturition behavior was measured using the automated voided stain on paper (aVSOP) method. Briefly, laminated filter paper pre-treated to turn deep purple in the presence of urine was continuously passed under a wire mesh at a rate of 10 cm per hour. Urine stains were converted to urinary output using the formula for the standard curve, calculated by the correlation of normal saline and the stained area ranging from 10–800 μl. The averages of 8 total hours (4 h before and after) were graphed starting from the beginning of the dark period. The amount of volume voided per micturition was averaged as the amount of volume voided per micturition over the elapsed time. The amount of voided volume per hour was calculated by dividing the amount of total voided volume by the elapsed time^6^.

### RNA-seq analysis

RNA conditioning, analysis, and normalization were performed according to Nagumo et al.^65^. Sequencing reads were mapped on the mm10 mouse reference genome, quantified, and then normalized with the quantile method using CLC Genomics Workbench version 10.1.1 (Qiagen). Circadian-oscillated genes, defined as MaxCorr to the cosine fit greater than 0.85 with an amplitude greater than 1.5-fold, were selected according to the method as previously described^34^. Enrichment Analysis were using Metascape. RNA-seq data are available at GSE205971.

### Activity recording

Mice were individually placed in plastic cages with a rotating exercise wheel (Melquest, Toyama, Japan)^66^. A revolution sensor was installed in the exercise wheel to detect spontaneous movements of the animals. Animals were caged for 7 days prior to the measurement and acclimatized so that the number of rotations would be constant.

### Statistics and reproducibility

For bladder mRNA expression, we used two-way repeated-measures ANOVA with Bonferroni’s post hoc test to compare differences among time points in the two groups of mice and one-way ANOVA with Dunnett’s multiple comparisons test to compare differences among three groups of mice at ZT12. Two-way repeated-measures ANOVA with Sidak’s and Dunnett’s multiple comparisons test were applied for the micturition experiments and Student’s t-test was used for the comparison of serum corticosterone levels. GraphPad Prism8 (GraphPad Software, San Diego, CA) was used for the statistical analyses. The sample size and number of replicates for each experiment are noted in their respective experimental sections. Data shown represent the results from *N* = 3–5 and two repeats of the same experiment.

## Supplementary information

supplementary information

Supplementary Data

## Data Availability

The source data underlying Figs. 1, 2, 3, 4, and 5 are provided as Supplementary Data. All data analyzed for RNA-seq comprising Figs. 5c, and 7 are available at GSE205971. A summary of data on the rhythm of volume voided per micturition in Fig. 6 is provided as Supplementary Tables S2 and S3. Other relevant data will be available upon reasonable request.
