# Supplementary material for: Glucocorticoids coordinate the bladder peripheral clock and diurnal micturition pattern in mice
Source: Commun Biol. 2023 Jan 21;6:81. doi: 10.1038/s42003-023-04464-3 (PMC9867708; doi:10.1038/s42003-023-04464-3)
Supplement: Supplementary file 1 — supplementary information [file 42003_2023_4464_MOESM1_ESM.pdf]

Supplementary Information

Glucocorticoids coordinate the bladder peripheral clock and diurnal micturition pattern in mice

Ichiro Chihara[1], Hiromitsu Negoro[1], Jin Kono[2], Yoshiyuki Nagumo[1], Haruki Tsuchiya[1], Kosuke Kojo[1], Masanobu Shiga[1], Ken Tanaka[3], Shuya Kandori[1], Bryan J. Mathis[4], Hiroyuki Nishiyama[1]

[1]Department of Urology, Faculty of Medicine, University of Tsukuba, Tsukuba, Ibaraki, Japan

[2]Department of Urology, Graduate School of Medicine, Kyoto University, Sakyo, Kyoto, Japan

[3]Department of Urology, Tsukuba Medical Center Hospital, Tsukuba, Ibaraki, Japan

[4]International Medical Center, University of Tsukuba Affiliated Hospital, Tsukuba, Ibaraki, Japan

Correspondence should be addressed to Hiromitsu Negoro (E-mail: hnegoro@md.tsukuba.ac.jp)

Supplementary Information includes:
Supplementary Figures S1 to S5
Supplementary Table S1 to S3

Supplementary Figure S1

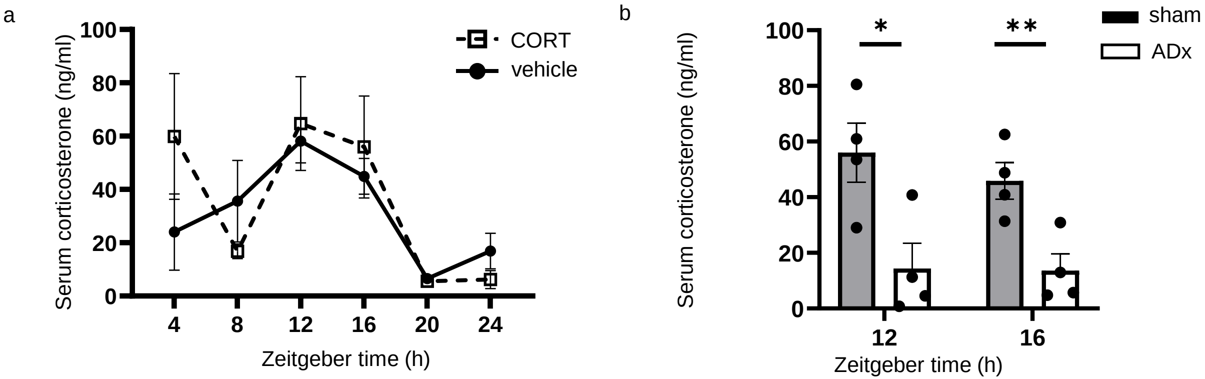

Supplementary Figure S1: Serum corticosterone level of CORT or ADx.
a Diurnal change in serum corticosterone levels in the vehicle and CORT groups. Each data series represents the mean ± SEM and 3 mice per time point. **b** Serum corticosterone levels at ZT12 and ZT 16 in the sham and ADx groups. Each data series represents the mean ± SEM and 4 mice per time point. Differences were determined by unpaired T test. *p<0.05, ** p<0.01 v.s. sham.

Supplementary Figure S2

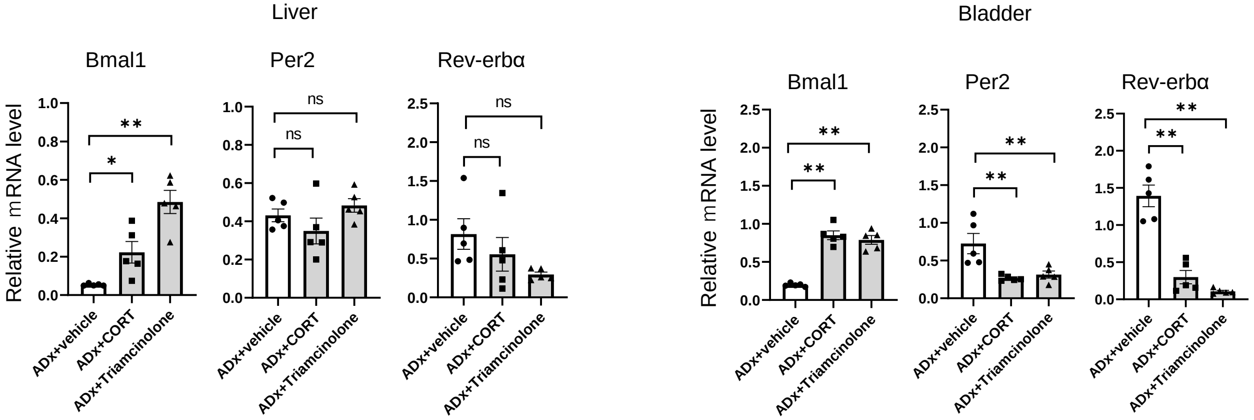

Supplementary Figure S2: Effect of triamcinolone administration.
Relative mRNA accumulation of *Bmal1*, *Per2* and *Rev-erbα* in the liver and bladder from ADx + vehicle, ADx + CORT and ADx + Triamcinolone at ZT 12. Each data series represents the mean ± SEM and 5 mice per time point. Differences were determined by one way ANOVA with Dunnett's multiple comparisons test. *p<0.05, * * p<0.01 v.s. ADx + vehicle.

Supplementary Figure S3

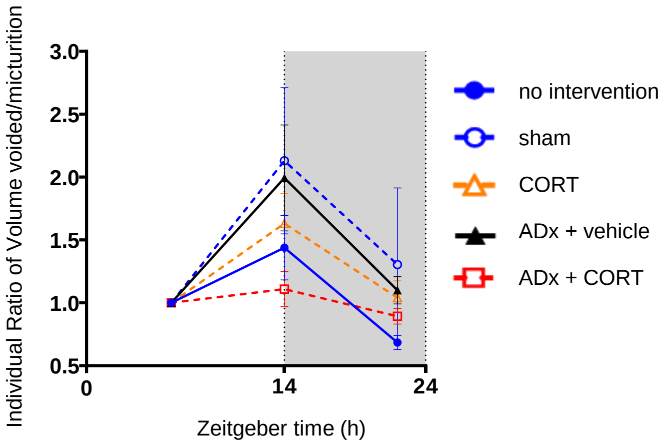

Supplementary Figure S3: The diurnal rhythm of micturition.
Average of 3 days results of measured volume voided per micturition average per 8-hour in each group, no intervention (no intervention; circle and solid blue line), adrenal sham surgery only (sham; circle and dash blue line), no surgery + corticosterone administration at ZT 1 (CORT; triangle and dash yellow line), bilateral adrenalectomy + methylcellulose administration at ZT 1 (ADx + vehicle; triangle and solid black line), bilateral adrenalectomy + corticosterone administration at ZT1 (ADx + CORT; square and dash red line). Each data series represents the ratio of ZT14, 22 to ZT6 as 1 in the same and represents the mean ± SEM and 3-5 mice per time point.

Supplementary Figure S4

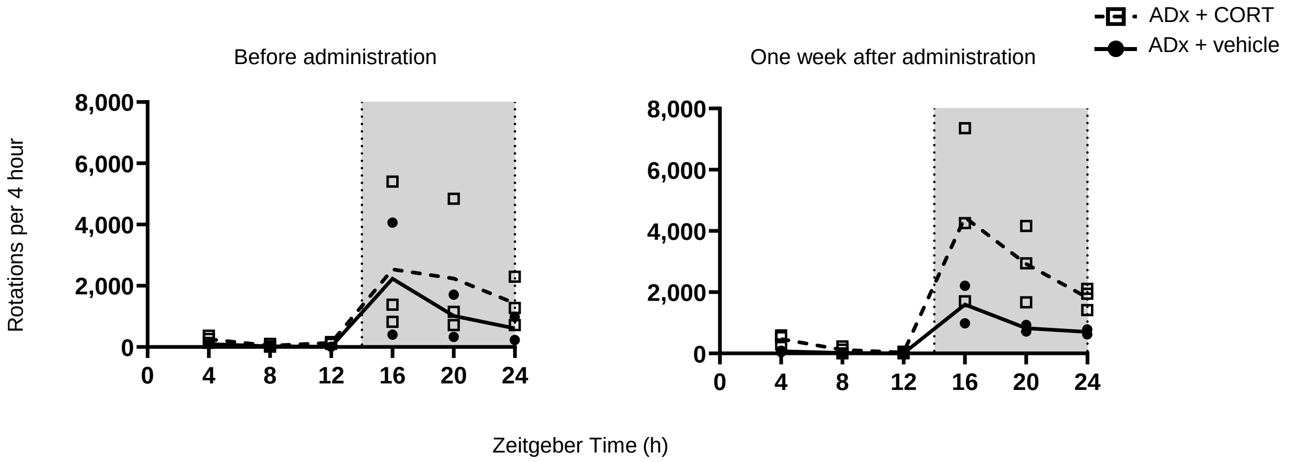

Supplementary Figure S4: Activity levels of ADx with vehicle or ADx with CORT administration at the non-physiological timing.

Diurnal changes in the number of spinning wheel rotations per 4 hours in the ADx + vehicle and ADx + CORT mice before corticosterone administration and after 7 days of corticosterone administration. Each data series represents the mean and 2 mice in the ADx + vehicle group and 3 mice in the ADx + CORT group.

Supplementary Figure S5

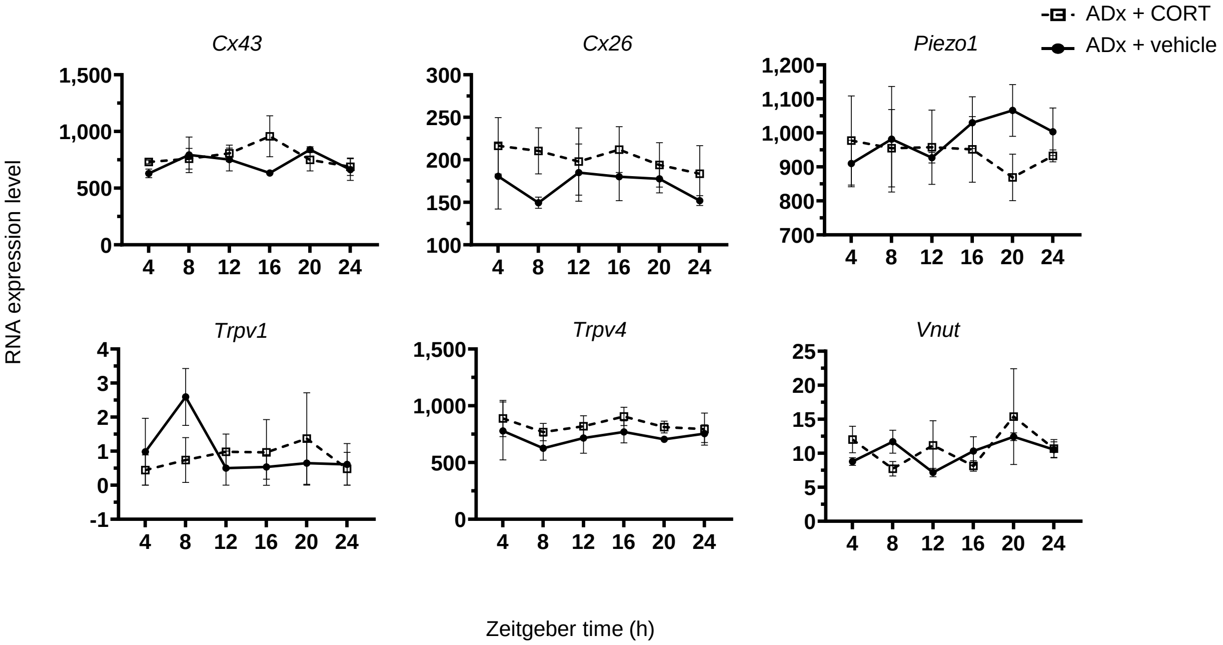

Supplementary Figure S5: Effects of bilateral adrenalectomy and non-physiologic corticosterone administration on gene expression known to be related to urinary function.
Temporal mRNA expression levels of genes known to be involved in urinary function by RNA-seq analysis in the bladder of ADx + vehicle (solid line) and ADx + CORT (dash line). Each data series represents 3 mice per time point.

Supplementary Table S1

a

gene name	ADx + vehicle MaxCorr	ADx + CORT MaxCorr	phase shift (forward)
Nr1d2	0.943717098	0.746900058	12
Nr1d1	0.983538826	0.69348514	8
Bhlhe41	0.856372285	0.682670482	12
Dbp	0.89258188	0.663353783	12
Bhlhe40	0.615353251	0.650542551	8
Per3	0.87594494	0.634540968	8
Tef	0.9133943	0.632538186	12
Arntl	0.956078695	0.625848101	12
Npas2	0.783181111	0.610863319	12
Per1	0.86431548	0.516769251	NA
Nfil3	0.835442491	0.507957032	NA
Cry2	0.915837366	0.498443072	NA
Cry1	0.945406533	0.487138792	NA
Per2	0.962302298	0.478160128	NA
Clock	0.837178295	0.340657461	NA
Rora	0.308317513	0.229215279	NA

b

gene name	Maxcorr	amplitude
Adamts12	0.86285102	2.489353891
Angptl7	0.862225445	12.19573835
Arntl	0.956078695	4.88419504
Bhlhe41	0.856372285	2.692755102
Cdc25b	0.888158177	1.656621865
Chodl	0.86481049	4.27807562
Clca3a2	0.859561695	2.756934616
Col17a1	0.855554978	2.610792377
Col5a3	0.884780878	2.327006695
Cry1	0.945406533	2.467813837
Dbp	0.89258188	10.09002877
Diras2	0.885827396	3.821609195
Lonrf3	0.872754037	2.682066703
Mfap2	0.852833885	2.073149365
Mt2	0.858458337	2.564239698
Mthfd1l	0.884590783	2.271023547
Naip6	0.876727526	1.819515194
Nr1d1	0.983538826	9.862147052
Nr1d2	0.943717098	2.540831785
Per1	0.86431548	2.929582772
Per2	0.962302298	4.026137595
Per3	0.87594494	3.349400317
Pitpnm2	0.864955793	1.909241089
Pxdn	0.859404749	1.725769058
Rasl11a	0.85932567	2.244349528
Tef	0.9133943	2.734162068
Thra	0.904728178	1.881161506
Tspan4	0.909598049	2.249201278
Wee1	0.931161903	1.749204907

Supplementary Table S1: Diurnal rhythmicity genes and clock genes in the ADx + vehicle and ADx + CORT groups.
a MaxCorr changes for clock genes in the ADx + vehicle and ADx + CORT groups. Clock genes are listed in descending order of MaxCorr value of ADx + CORT and peak time shifts with MaxCorr greater than 0.6 are also listed. (NA: not analyzed) **b** MaxCorr and amplitude of expression level for 29 diurnal rhythmicity genes (defined as greater than the max correlation of 0.85 from the cosine curve with a 1.5-fold amplitude of expression level) in the ADx + vehicle group.

Supplementary Table S2

a

No.									
1		2		3		4		5	
ZT	Volume voided /micturition (μl)	ZT	Volume voided /micturition (μl)	ZT	Volume voided /micturition (μl)	ZT	Volume voided /micturition (μl)	ZT	Volume voided /micturition (μl)
4.9	568.2	3.2	341.8	3.7	158.8	4.7	326.7	4.8	252.8
13.1	352.9	3.9	249.7	15.9	642.5	10.0	498.1	14.8	644.2
20.5	350.7	11.1	179.4	18.5	446.4	15.8	589.8	18.9	440.9
23.1	179.8	27.8	580.3	20.3	215.3	17.9	402.7	23.2	537.3
33.8	114.0	33.9	296.1	23.6	267.2	20.3	456.0	23.9	192.3
53.5	17.9	38.4	200.1	25.2	171.9	22.0	313.0	27.3	339.3
61.8	160.2	43.3	185.0	28.3	139.5	23.3	334.3	31.5	417.4
63.7	276.4	50.4	158.5	41.0	595.6	25.1	233.6	39.8	753.9
73.5	241.9	54.6	272.0	43.2	343.0	26.7	265.5	42.8	432.2
		56.6	9.3	45.1	19.0	28.4	250.5	46.0	492.9
		61.5	590.2	45.4	238.9	31.9	470.3	47.8	314.9
		63.4	433.1	49.0	204.5	37.0	600.1	53.5	574.7
		65.0	354.8	51.6	193.8	40.2	323.3	60.2	781.4
		66.8	389.3	65.2	415.5	42.8	283.4	65.3	493.7
		72.3	801.6	66.7	201.1	42.1	149.3	66.0	331.3
				67.7	124.9	43.5	261.3	70.1	460.1
				69.3	273.9	44.2	134.2	70.6	394.6
				71.7	214.0	46.5	407.6	71.7	332.5
						48.1	329.4		
						49.6	288.0		
						50.0	209.2		
						53.3	422.0		
						59.8	584.4		
						63.9	488.9		
						66.0	414.6		
						67.1	319.7		
						68.4	241.4		
						71.0	487.1		
						73.0	316.0		

b

No.									
1		2		3		4		5	
ZT	Volume voided /micturition (μl)	ZT	Volume voided /micturition (μl)	ZT	Volume voided /micturition (μl)	ZT	Volume voided /micturition (μl)	ZT	Volume voided /micturition (μl)
3.8	315.2	5.9	329.6	3.7	392.2	3.1	270.7	2.5	491.2
8.6	395.8	16.1	9.0	10.6	455.1	4.0	214.0	4.3	325.8
17.6	930.6	19.2	78.1	16.8	335.4	6.8	330.1	14.7	679.8
20.3	316.7	20.2	265.1	17.5	232.0	14.6	576.5	17.8	373.3
22.5	212.5	23.2	451.9	19.2	188.7	16.2	353.9	19.5	342.7
24.0	138.8	25.5	192.3	21.8	153.2	18.0	336.2	22.1	295.3
26.4	375.8	26.9	138.7	24.6	210.4	22.0	70.5	23.9	141.5
42.0	907.2	31.7	85.2	26.2	273.5	23.2	372.8	27.5	282.2
44.7	426.2	35.2	422.7	32.0	359.1	25.0	285.0	34.0	318.4
47.0	199.9	41.1	118.2	39.1	345.8	26.6	376.9	40.2	542.2
48.1	403.1	41.9	179.2	41.7	224.7	28.5	284.1	42.4	334.4
65.1	983.8	43.0	89.3	43.3	302.6	30.1	374.3	43.7	288.9
68.9	583.2	44.1	456.6	45.6	299.6	33.5	417.3	46.5	330.8
71.7	327.7	47.4	202.9	47.8	254.3	38.1	434.9	47.4	210.6
73.7	295.6	49.6	320.7	50.6	176.9	40.3	444.7	50.6	430.4
		51.7	104.7	63.4	411.6	41.9	328.8	54.2	477.2
		56.2	385.5	64.8	377.7	43.8	248.7	58.0	345.0
		64.9	139.3	66.9	192.1	46.2	283.6	64.3	605.7
		66.5	94.4	67.5	147.5	47.0	303.4	66.6	377.7
		66.9	161.0	69.5	162.1	47.8	174.2	68.2	366.9
		67.7	41.6	71.8	225.8	50.3	338.6	71.4	343.8
		68.3	315.7	73.3	149.0	52.1	349.3		
		69.0	54.5			52.8	363.8		
		69.4	10.7			53.3	207.0		
		69.6	45.4			55.0	227.8		
		70.8	263.3			58.2	347.7		
		73.4	217.8			58.5	357.7		
						62.5	508.4		
						64.6	520.8		
						67.2	568.2		
						70	365.48		
						70.3	180.99		
						71.6	366.77		
						73.4	270.83		

Supplementary Table S2: All data of volume voided per micturition for 3 consecutive days.
All data of volume voided per micturition measured by aVSOP method for 3 consecutive days in No.1 to 5 mice of ADx + vehicle (a) and of ADx + CORT (b).

Supplementary Table S3

		no intervention			Sham			CORT			ADx+vehicle			ADx+CORT		
**ZT**		**6**	**14**	**22**	**6**	**14**	**22**	**6**	**14**	**22**	**6**	**14**	**22**	**6**	**14**	**22**
No.	1	1	0.98	0.57	1	1.89	0.73	1	2.09	1.18	1	2.49	0.83	1	1.22	1.09
	2	1	1.87	0.76	1	3.24	2.52	1	1.30	0.77	1	0.95	1.33	1	0.57	0.94
	3	1	1.47	0.72	1	1.26	0.66	1	1.50	1.17	1	3.36	1.38	1	1.15	0.73
	4										1	1.48	0.98	1	1.35	0.91
	5										1	1.69	0.98	1	1.25	0.80

Supplementary Table S3: The diurnal rhythm of volume voided per micturition.
Average of 3-day data of 8-hourly averages of volume voided per micturition in each group, no intervention (no intervention), sham (adrenal sham surgery only), CORT (no surgery + corticosterone administration at ZT 1), ADx + vehicle (bilateral adrenalectomy + methylcellulose administration at ZT 1), ADx + CORT (bilateral adrenalectomy + corticosterone administration at ZT1). Each data sets represents the ratio of ZT14, 22 to ZT6 as 1 in the same mice and 3-5 mice per group.
